# Prediction of distinct populations of innate lymphoid cells by transcriptional profiles

**DOI:** 10.3389/fgene.2023.1227452

**Published:** 2023-08-31

**Authors:** Haiyao Dong, Zhenguang Du, Haoming Ma, Zhicheng Zhou, Haitao Yang, Zhenyuan Wang

**Affiliations:** ^1^ Department of Thoracic Surgery, China Medical University, Shenyang, China; ^2^ Department of Thoracic Surgery, The People’s Hospital of Liaoning Province, Shenyang, China; ^3^ Department of No. 3 Oncology, The People’s Hospital of Liaoning Province, Shenyang, China; ^4^ College of Software, Northeastern University, Shenyang, China

**Keywords:** innate lymphoid cells, machine learning, DNN, LASSO, gene expression

## Abstract

Innate lymphoid cells (ILCs) are a unique type of lymphocyte that differ from adaptive lymphocytes in that they lack antigen receptors, which primarily reside in tissues and are closely associated with fibers. Despite their plasticity and heterogeneity, identifying ILCs in peripheral blood can be difficult due to their small numbers. Accurately and rapidly identifying ILCs is critical for studying homeostasis and inflammation. To address this challenge, we collect single-cell RNA-seq data from 647 patients, including 26,087 transcripts. Background screening, Lasso analysis, and principal component analysis (PCA) are used to select features. Finally, we employ a deep neural network to classify lymphocytes. Our method achieved the highest accuracy compared to other approaches. Furthermore, we identified four genes that play a vital role in lymphocyte development. Adding these gene transcripts into model, we were able to increase the model’s AUC. In summary, our study demonstrates the effectiveness of using single-cell transcriptomic analysis combined with machine learning techniques to accurately identify congenital lymphoid cells and advance our understanding of their development and function in the body.

## Highlights

• Our study demonstrates the feasibility of combining machine learning methods with feature extraction models for cell immunotyping.
• To compare various classification models and feature extraction methods, we conducted comparative experiments and determined that the optimal model was the combination of DNN and LASSO.
• Our findings indicate that the incorporation of the four genetic information found in the literature can enhance the accuracy of the classification model.

## 1 Introduction

In the past decade, innate lymphoid cells (ILCs) have garnered significant attention from researchers due to their crucial role in the innate immune system (Hazenberg and Spits, 2014; Artis and Spits, 2015; Eberl et al., 2015; Vivier et al., 2018). These heterogeneous lymphocytes originate from lymphoid progenitor cells in the bone marrow. Notably, they lack the rearrangement of antigen-specific receptors that depend on recombination activation genes, do not express antigen-specific receptors unique to acquired immune cells, and do not exhibit surface markers similar to those found on other immune cells. The transcription factors and secreted cytokines necessary for the development of various ILCs differ. Based on these factors and cytokines, ILCs can be classified into natural killer (NK) cells, ILC1s, ILC2s, ILC3s, and others (Montaldo et al., 2015; Miller et al., 2018; Vacca et al., 2019). ILCs are predominantly found in barrier regions such as the respiratory tract, digestive tract mucosa, and skin. They respond to local cytokine signals in their microenvironment and serve early immune surveillance and regulatory functions by secreting cytokines and other mediators. ILCs also act as a bridge between innate and acquired immunity, regulating systemic immune responses by coordinating the functions of acquired immune cells.

The accurate classification of ILCs is of great medical significance, as the functions and behaviors of various ILCs in the body can vary greatly. Abnormalities in the functions of ILCs can impact the onset and progression of various conditions, including inflammation, autoimmune diseases, metabolic disorders, and allergies (Elemam et al., 2017; Li et al., 2017; Golebski et al., 2019; Bartemes and Kita, 2021; Kabata et al., 2022; Kumar, 2022; Surace and Wilhelm, 2022). To effectively utilize the potential of ILCs in disease diagnosis and treatment, it is crucial to classify ILCs with the highest possible accuracy. Misclassification can lead to the use of inappropriate treatment strategies in clinical trials (Everaere et al., 2016; Everaere et al., 2018; Kogame et al., 2022). Despite their significant role, there is currently no effective method for identifying different cell subpopulations among ILCs. At present, the identification of ILCs primarily rely on flow cytometry, which uses specific antibodies to identify surface markers of different types of ILCs. However, as there are many types of ILCs, there often be some overlap in the functions of each subgroup. Therefore, the accuracy of marker-based identification techniques needs to be improved. In addition, although new sequencing technologies are developing (Hu et al., 2023), revealing patterns of gene expression at the cellular level, these new sequencing technologies are often costly. All in all, there is a need for accurate and low-cost methods for identifying ILCs.

In recent years, computational methods have been widely applied to mine biological information using omics data (Tyanova et al., 2016; Hériché et al., 2019; Efremova and Teichmann, 2020; Kaur et al., 2021; Badia-i Mompel et al., 2022; Watson et al., 2022). The expansion of genomic, proteomic, transcriptomic, and metabolomic data has provided an amazing opportunity for the application of machine learning methods. Researchers have developed a large number of tools, methods, and resources to fully utilize these data for precision medicine.

In this paper, we obtained genes associated with innate immune cells and used their expression levels to predict immune typing. Differential gene expression levels can directly reflect the developmental state of cells, and changes in gene expression can also affect the levels of proteins and metabolites. This work has yielded a precise and low-cost approach to classifying ILCs.

## 2 Methods

In this section, we provided a detailed overview of the implementation of this work, which includes the framework, data preprocessing, feature compression, and evaluation. To construct our machine learning model, we needed three main components: data points, features, and labels (Jung, 2022). In this study, we built our model using the expression matrix of innate immune cells obtained from the GEO database, where data points represent individual cells, features represent the intensity of gene expression, and labels indicate the immune subtype that each cell corresponds to. With these components, we were able to develop and evaluate our machine learning models.

### 2.1 Workflow

**FIGURE 1:**
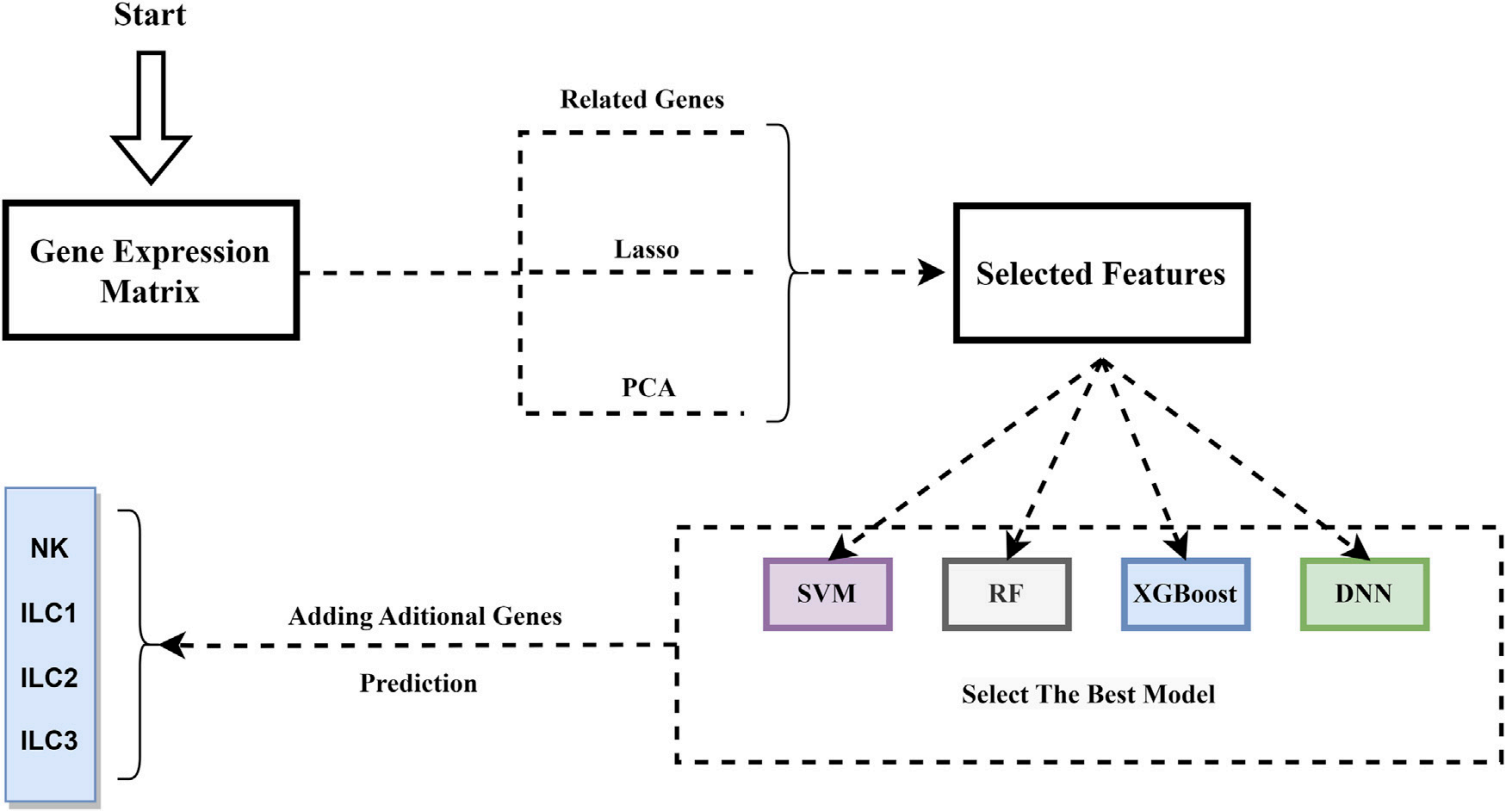
The framework of our work.

Firstly, we obtained single-cell transcriptome data associated with innate lymphoid cells from the GEO database (Barrett et al., 2012), and then obtained known genes associated with innate lymphoid cells from DisGeNet (Piñero et al., 2020). We performed Related genes, PCA and LASSO analysis using the expression of genes associated with innate lymphoid cells as features and extracted the most significant features related to immune typing. In addition, in their latest study, Korchagina et al. identified STATS, BATF, IKAROS, RUNX3, C-MAF, BCL11B, and ZBTB46 as genes closely associated with Innate Lymphoid Cells. And STATS, IKAROS, and C-MAF have been included already in our previous gene set. Therefore, we incorporated the expression of four additional hub genes in our subsequent analysis. After feature dimensionality reduction, we inputted all these features into a deep neural network (DNN). The DNN displayed immune typing based on important gene expression features. The workflow of our method is shown in Figure 1.

### 2.2 Dateset processing

For our study, we utilized the Bjroklund dataset (Björklund et al., 2016), which comprises sequencing data of lymphoid cells from three independent human volunteers. We conducted a comprehensive search of ILCs-related datasets across multiple databases. Unfortunately, many of these datasets were incomplete, with inadequate or absent classification of ILCs. However, the Bjorklund dataset, which is from human tonsil, emerged as a standout resource. Published in Nature Immunology, it has been widely cited and is considered authoritative. Therefore, we chose it to performe downstream analysis. In this dataset, the specimens for transcriptional analysis came from three donors: a 56-year-old (Donor A), a 44-year-old (Donor B), and a 23-year-old (Donor C). All procedures were carried out at the Karolinska University Hospital, located in Huddinge. The regional ethical committee at the Karolinska Institutet granted approval for the collection of these anonymous tissue samples. Informed consent was provided by all patients, or by their legal guardians in cases where the patients were under 18 years of age. We selected 647 samples annotated with immune typing, including 74 NK cells, 126 ILC1 cells, 139 ILC2 cells, and 308 ILC3 cells. Each sample in this dataset contains 26,087 transcripts. We mapped these transcripts to their corresponding genes using the GRCh37 assembly of the human genome. Additionally, we obtained 292 genes associated with innate lymphoid cells from DisGeNet. Moreover, our analysis of additional studies revealed that BCL11B, BATF, RUNX3, and ZBTB46 are associated with lymphoid cell immune typing (Korchagina et al., 2023).

### 2.3 Feature compression

This section presents a comparative analysis of three commonly used feature compression methods. For each method, we provide detailed information about its implementation.

• Related Genes

To reduce the dimensionality of features using the related gene method, we followed a specific process. First, we obtained a gene set related to the disease from the DisGeNet website. Then, we intersected this gene set with the gene set of the original data to obtain a new, reduced gene set.

• PCA

Principal component analysis (PCA) is a commonly used data dimensionality reduction technique, which aims to transform high-dimensional data into lower-dimensional data for better data processing and analysis (Daffertshofer et al., 2004). The basic idea of PCA is to map the original data to a new coordinate system via linear transformation, such that the mapped data has the maximum variance. This linear transformation is achieved by computing the covariance matrix of the data and its eigenvectors. Specifically, assume that we have an *n* × *p* data matrix *X*, where *n* is the number of samples and *p* is the number of variables. We first need to center *X* by subtracting the mean of each variable from the entire variable, resulting in a new matrix $\tilde{X}$ , where,

$${\tilde{X}}_{ij}=X_{ij}-\frac{1}{n}\overset{n}{\underset{k=1}{\sum }}X_{kj}$$ (1)

Next, we compute the covariance matrix *S* of $\tilde{X}$ , i.e.,

$$S=\frac{1}{n-1}{\tilde{X}}^{T}\tilde{X}$$ (2)

Then, we perform eigendecomposition on *S* to obtain the eigenvalues *λ*_1_ ≥ *λ*_2_ ≥⋯ ≥ *λ*_*p*_ and their corresponding eigenvectors *v*_1_, *v*_2_, *…*, *v*_*p*_. Finally, we select the top *k* eigenvectors *v*_1_, *v*_2_, *…*, *v*_*k*_ and project the data *X* onto these eigenvectors to obtain a new *n* × *k* matrix *Y*, where $Y_{ij}=v_{j}^{T}X_{i}$ . In this way, we complete PCA dimensionality reduction, transforming the original *n* × *p* data matrix *X* into a new *n* × *k* matrix *Y*, where *k* is the number of variables we choose.

• LASSO

Lasso (Least Absolute Shrinkage and Selection Operator) is a widely used linear regression technique for feature selection and sparse modeling (Osborne et al., 2000). Lasso constrains the model parameters using L1 regularization, which shrinks some parameters to zero, achieving feature selection. Specifically, we can represent Lasso regression using the following formula:

$$\underset{\beta }{\min}\frac{1}{2n}\|y-X\beta {\|}_{2}^{2}+\lambda \|\beta {\|}_{1}$$ (3)

Here, *β* is the model parameter vector, *X* is the feature matrix, *y* is the response variable vector, and *λ* is the regularization parameter. The L1 regularization term ‖*β*‖_1_ shrinks some parameters to zero, achieving feature selection and model sparsity. By adjusting the regularization parameter *λ*, we can control the degree of sparsity of the model.

### 2.4 DNN four-class classification

A Four-class deep neural network (DNN) can be defined as a function $f:R^{n}\to R^{4}$ that maps an input vector $x\in R^{n}$ to an output vector $y\in R^{4}$ . This function can be represented as a composition of layers, where each layer *i* consists of a linear transformation ***z*** ^(*i*)^ = ***W*** ^(*i*)^ ***h*** ^(*i*−1)^ + ***b*** ^(*i*)^ and a nonlinear activation function *g* ^(*i*)^, with ***h*** ^(0)^ = ***x*** as the input and ***h*** ^(*i*)^ = *g* ^(*i*)^(***z*** ^(*i*)^) as the output of layer *i*. The weights and biases of each layer are denoted by ***W*** ^(*i*)^ and ***b*** ^(*i*)^, respectively. The process can be formulated as:

$$z^{\left(L\right)}=W^{\left(L\right)}h^{\left(L-1\right)}+b^{\left(L\right)}$$ (4)

In the last layer, we apply the softmax function, as below:

$$y=\text{softmax}\left(z^{\left(L\right)}\right)$$ (5)

To transform the output ***z*** ^(*L*)^ into a probability vector ***y***, where *L* is the index of the last layer. During training, we use the cross-entropy loss function, as:

$$L\left(y,t\right)=-\overset{4}{\underset{i=1}{\sum }}t_{i}\log y_{i}$$ (6)

To measure the difference between the predicted probability vector ***y*** and the true label vector ***t***, where *t*_*i*_ ∈ {0, 1} indicates whether the *i*-th class is the correct class.

The training process we use backpropagation to compute gradients and update weights and biases. At each training iteration, we feed the input vector ***x*** to the DNN, compute the output vector ***y***, compare it with the true label vector ***t***, then use backpropagation to compute the gradients and use optimization algorithms such as gradient descent to update weights and biases.

### 2.5 Evaluating metrics

Accuracy is a performance metric used to evaluate classification models, which represents the proportion of correctly classified samples to the total number of samples.

Specifically, given a classification model with predicted labels ${\hat{y}}_{i}$ and corresponding true labels *y*_*i*_, the accuracy of the model can be calculated using the following formula:

$$\text{Accuracy}=\frac{\sum_{i=1}^{n}1\left({\hat{y}}_{i}=y_{i}\right)}{n}$$ (7)

Here, *n* is the total number of samples, and 1 is the indicator function that takes the value 1 when ${\hat{y}}_{i}=y_{i}$ and 0 otherwise. The numerator of the formula represents the sum of correctly classified samples, and the denominator represents the total number of samples, i.e., the proportion of correctly classified samples to the total number of samples.

### 2.6 Parameter

To ensure the reproducibility of our experiments, we provide detailed information on the model parameters used in this work. We implemented PCA and Lasso dimensionality reduction methods using the sklearn library. To ensure fairness in dimensionality reduction, all three methods were reduced to the same dimension 200. So PCA was set 200 principal components, while the alpha value in the Lasso model was set to 0.1. For the four classification models, three machine learning models were built using sklearn library, while the DNN was designed manually. The network layer parameters were set to (512, 64, 4), and the learning rate was set to 0.001.

## 3 Results

In this section, we conducted a thorough analysis of the sample size to ensure its appropriateness. Subsequently, we evaluated various methods for feature dimensionality reduction and machine learning classification, and selected the optimal model. Finally, we examined the impact of incorporating hub genes and validated their effectness.

### 3.1 Sample size verification

Our analysis involved raw data from the GEO database, which included properties of immune typing. To ensure clear labeling, we assigned the labels 0, 1, 2, and 3 to NK cells, ILC1, ILC2, and ILC3, respectively. This resulted in a dataset containing four types of samples, consisting of 74 NK cells, 126 ILC1 cells, 139 ILC2 cells, and 308 ILC3 cells. Importantly, the sample was relatively balanced among the different types of cells.

To assess the statistical performance of our data, we used an online web server called SSizer (Li et al., 2020). And the result illustrates that our data met Type 3 statistical indicators, indicating that our sample size was sufficient for our analysis. Furthermore, this demonstrates that when the sample size exceeded 300, the overlap was 0.5, indicating that our sample size was appropriate.

### 3.2 Comparative results

To provide a comprehensive evaluation of our method, we compared it with several other methods. Since there is no consensus on the best feature selection method, we simultaneously compared three different feature selection methods. The feature selection process is described in detail in Section 2 of our study. In addition, our method is based on deep neural networks (DNN) and compared with several other common machine learning classification models, including support vector machines, XGBoost, and random forests (Wang and Hu, 2005; Chen et al., 2015; Biau and Scornet, 2016). We tested each classification model using the three methods of feature selection, resulting in a total of 12 groups of classification results.

**FIGURE 2:**
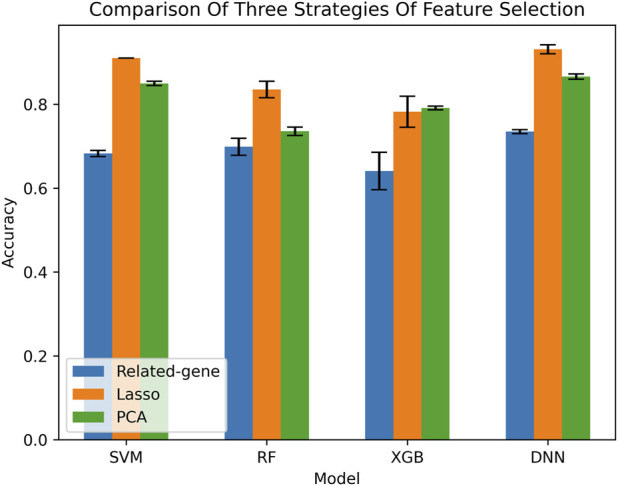
Comparison of DNN method with several other models by AUC.

To assess the accuracy of our method, we performed 5-fold cross-validation. This is a common machine learning model evaluation method where the dataset is randomly divided into five mutually exclusive subsets. Each subset is used once as a validation set while the remaining four subsets are used for training. This process is repeated five times, with each subset used once as the validation set, to evaluate the performance of the model. The results, as shown in Figure 2, unequivocally demonstrate that the optimal classification combination is achieved using the DNN + LASSO method. Our comparison analysis provides valuable insights into the effectiveness of different feature selection methods and classification models for our specific dataset, and our results can inform future research in this field.

**FIGURE 3:**
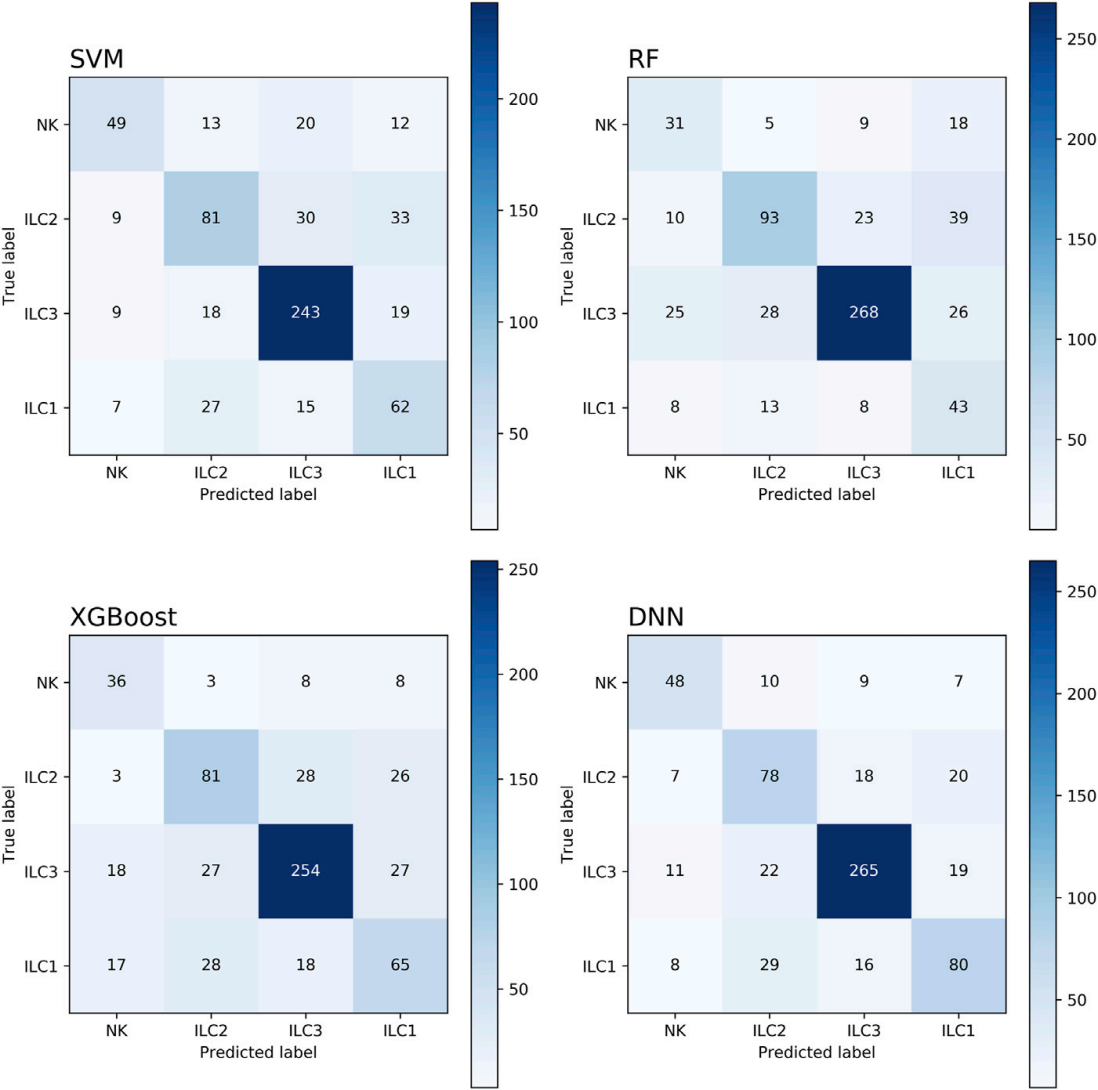
The confusion matrix of four models.

**FIGURE 4:**
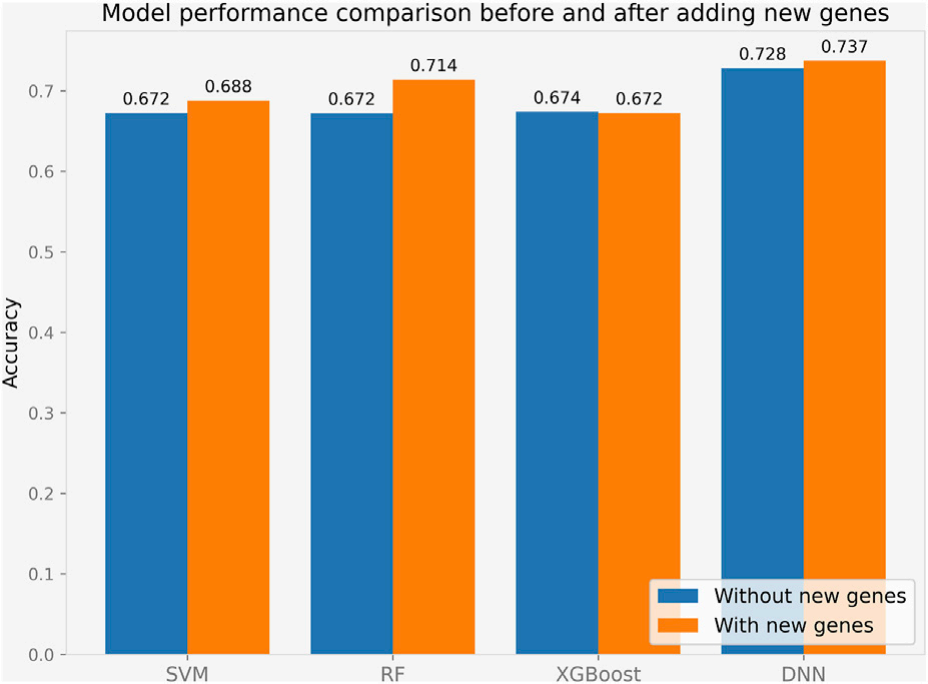
Model performance with or without four key genes.

Although other feature dimensionality reduction methods seem to achieve better performance, the related gene method provides an intuitive characterization. We further verified the performance of the model on the related gene method. The confusion matrices of different machine learning classification models are presented in Figure 3, which is a widely used tool for evaluating classification model performance and measuring prediction accuracy. A confusion matrix is a two-dimensional table that represents the true and predicted labels of a classification model, where the rows correspond to the true labels and the columns correspond to the predicted labels. Each element of the matrix represents the count of samples for which the classification model predicted the category shown in the corresponding column, while the actual category was shown in the corresponding row. Based on the results shown in Figure 4, it is evident that the DNN classification model outperforms the other models. These findings suggest that the DNN model may be a more effective approach for this specific classification task.

### 3.3 Model performance with key genes

We performed a literature investigation and identified four genes, BCL11B, BATF, RUNX3, and ZBTB46, that are associated with Innate Lymphoid Cells. To investigate whether transcripts of these genes could improve the prediction of immune typing, we incorporated them into our model. We conducted several experimental comparisons to fully evaluate the impact of these transcripts on the model’s accuracy, and the results are shown in Figure 4.

Our experiments demonstrated that the addition of these transcripts significantly enhanced the accuracy of the model. Among all the methods tested, LASSO-DNN showed the best performance. These findings suggest that incorporating the transcripts of these four genes, particularly in combination with LASSO-DNN, has the potential to improve the performance of the immune typing model. Therefore, our study highlights the importance of these genes in immune typing and provides a framework for future research in this field.

## 4 Conclusion

Cellular immunoassay detection currently relies on sequencing technology and biological experiments. With the continuous development of medicine, immunotherapy has significantly improved the survival rate of patients with advanced cancer. Therefore, it is of utmost clinical and basic research significance to predict immune typing in advance. To achieve this, two critical steps are necessary, namely, identifying key transcripts and building effective machine learning models to accurately predict immune typing based on these transcripts.

In this study, we identified genes associated with Innate Lymphoid Cells and obtained their corresponding transcription expression levels. We used three feature extraction methods for feature dimension reduction and designed a DNN model for predicting immune typing. We compared the performance of the DNN method with several other methods and found that the combination of DNN and LASSO provided the best classification performance. Moreover, we tested whether the information of four genes found in literature research could improve the accuracy of our model. These four genes provided valuable information and effectively enhanced the accuracy of the model in predicting immune typing.

While our method was developed specifically for the classification of ILCs, it has the potential to be applied in other contexts. In general, the machine learning model we have developed processes numerical matrices based on biological data from cells, and can produce results for any such dataset. However, one important factor to consider is the similarity between ILCs and other cell types. If other cell types share similar gene expression patterns with ILCs, our approach may also be effective for these cell types. Conversely, if the gene expression patterns are significantly different, our approach may not be suitable.

In summary, our study presents a novel method for predicting immune typing and demonstrates the accuracy of genes previously identified in literature research. Our findings contribute to the advancement of immune typing prediction and provide a framework for future research in this field.

## Data Availability

The original contributions presented in the study are included in the article/Supplementary Material, further inquiries can be directed to the corresponding authors.
